# Establishment of the basidiomycete *Fomes fomentarius* for the production of composite materials

**DOI:** 10.1186/s40694-022-00133-y

**Published:** 2022-02-24

**Authors:** Carsten Pohl, Bertram Schmidt, Tamara Nunez Guitar, Sophie Klemm, Hans-Jörg Gusovius, Stefan Platzk, Harald Kruggel-Emden, Andre Klunker, Christina Völlmecke, Claudia Fleck, Vera Meyer

**Affiliations:** 1 Chair of Applied and Molecular Microbiology, Technische Universität Berlin, Str. des 17. Juni 135, 10623 Berlin, Germany; 2 Chair of Materials Science and Engineering, Technische Universität Berlin, Str. des 17. Juni 135, 10623 Berlin, Germany; 3 Department of Post Harvest Technology, Leibniz-Institute for Agricultural Engineering and Bioeconomy (ATB), Max-Eyth-Allee 100, 14469 Potsdam, Germany; 4 Chair of Mechanical Process Engineering and Solids Processing (MVTA), Technische Universität Berlin, Str. des 17. Juni 135, 10623 Berlin, Germany; 5 Stability and Failure of Functionally Optimized Structures Group, Technische Universität Berlin, Str. des 17. Juni 135, 10623 Berlin, Germany

**Keywords:** Filamentous fungi, *Fomes fomentarius*, Circular economy, Bioeconomy, Hemp, Rapeseed, Composite material, Mycelium, Neo-Hooke model, Finite element method FEM, Mechanical properties, Compressive strength, Stiffness

## Abstract

**Background:**

Filamentous fungi of the phylum Basidiomycota are considered as an attractive source for the biotechnological production of composite materials. The ability of many basidiomycetes to accept residual lignocellulosic plant biomass from agriculture and forestry such as straw, shives and sawdust as substrates and to bind and glue together these otherwise loose but reinforcing substrate particles into their mycelial network, makes them ideal candidates to produce biological composites to replace petroleum-based synthetic plastics and foams in the near future.

**Results:**

Here, we describe for the first time the application potential of the tinder fungus *Fomes fomentarius* for lab-scale production of mycelium composites. We used fine, medium and coarse particle fractions of hemp shives and rapeseed straw to produce a set of diverse composite materials and show that the mechanical materials properties are dependent on the nature and particle size of the substrates. Compression tests and scanning electron microscopy were used to characterize composite material properties and to model their compression behaviour by numerical simulations. Their properties were compared amongst each other and with the benchmark expanded polystyrene (EPS), a petroleum-based foam used for thermal isolation in the construction industry. Our analyses uncovered that EPS shows an elastic modulus of 2.37 ± 0.17 MPa which is 4-times higher compared to the *F. fomentarius* composite materials whereas the compressive strength of 0.09 ± 0.003 MPa is in the range of the fungal composite material. However, when comparing the ability to take up compressive forces at higher strain values, the fungal composites performed better than EPS. Hemp-shive based composites were able to resist a compressive force of 0.2 MPa at 50% compression, rapeseed composites 0.3 MPa but EPS only 0.15 MPa.

**Conclusion:**

The data obtained in this study suggest that *F. fomentarius* constitutes a promising cell factory for the future production of fungal composite materials with similar mechanical behaviour as synthetic foams such as EPS. Future work will focus on designing materials characteristics through optimizing substrate properties, cultivation conditions and by modulating growth and cell wall composition of *F. fomentarius*, i.e. factors that contribute on the meso- and microscale level to the composite behaviour.

**Supplementary Information:**

The online version contains supplementary material available at 10.1186/s40694-022-00133-y.

## Introduction

Fungal biotechnology is an innovation driver for the bioeconomy with its principles of circular economy and sustainability [1, 2]. Especially filamentous fungi have a rich and very versatile metabolism that forms the basis for a diverse palette of products, which become harnessed by the food, beverage, pharmaceutical, biofuel, textile, feed, automotive, packaging and chemical industries. However, filamentous fungi are not only masters of biosynthesis, they are also masters of decomposition. Their ability to degrade and transform lignocellulosic substrates into composite materials is unique in nature and attracted a lot of interest recently [1, 2]. In several interdisciplinary endeavours, fungal bio(techno)logists, designers, process engineers and material scientists have collaborated to turn by-products from agriculture and forestry with the help of basidiomycetes into composite materials as highlighted in recent reviews [3–5]. The vision is surprising and fascinating, yet plausible and thus hopefully achievable in the near future: Plastics, foams, textiles and other materials derived from petroleum-based resources could soon be functionally replaced by a new class of biomaterials produced by fungal biotechnology [2]. Given the urgent need to reduce global carbon dioxide emission and plastic pollution, the pressure to innovate is indeed high. Within the last 5 years, the ability of fungal mycelium not only to digest but also to bind and connect loose plant-based particles into firmer composite materials has thus led to a substantial increase in publications that pioneered the manufacturing process and that described some characteristics of mycelium-based materials [6–12]. Potential applications for fungal composite materials that have been discussed so far are as diverse as disruptive—soon packaging material, thermal insulation, acoustic insulation, construction material as well as leather could be produced by filamentous fungi of the phylum Basidiomycota [2–4, 13–15].

To contribute to these research efforts, we ran a bioprospecting program in 2018 in our Berlin-Brandenburg area to explore the local biodiversity of mushroom-forming fungi and to build up a strain collection of basidiomycetes that reflects the predominant regional biodiversity and that feeds well on regional renewable plant resources. As recently described [16], we could isolate and identify about 75 basidiomycetes, most of which were assigned to the order *Polyporales*, including the tinder fungus *Fomes fomentarius*, the fire sponge *Phellinus robustus*, *Ganoderma adspersum*, the artist´s bracket *Ganoderma applanatum* and the turkey tail *Trametes versicolor*. Also, representatives of the order *Agaricales* became members of the strain collection including the oyster mushroom *Pleurotus ostreatus*, the stump mushroom *Armillaria ostoyae* and the similar looking *Pholiota limonella* [16]. In growth experiments on different substrates from regional agricultural residual streams, the white-rot fungi *F. fomentarius*, *P. ostreatus* and *T. versicolor* excelled with the best performance [16].

Various considerations let us to focus our further research on the tinder fungus *F. fomentarius*. This basidiomycete, which is prevalent throughout the temperate climate zone of the northern hemisphere, is well-known to traditional medicine and thus has a rich ethnomycological tradition [17, 18]. Furthermore, the trama of its fruiting bodies has been safely used by mankind for hundreds of years as wound dressing and leather alternative [19]. Remarkably, the fruiting bodies are water-repellent, very stable and light-weighted. Interestingly, the hymemium follows a hierarchically honeycomb structure and was previously already subjected to mechanical testing [20], showing compressive stress–strain curves of foams, where an initially linear course is followed by an extended plateau region [20]. Given that such characteristics could be adjusted in the future for laboratory cultivated *F. fomentarius* mycelia that were fed on renewable plant biomass, new materials for lightweight applications, specifically for anisotropic loading conditions could be developed. Finally, the genome sequence of one *F. fomentarius* isolate identified in France has been recently published (strain CIRM-BRFM 1821) [21] and uncovered many genes in its genome predicted to encode lignin-active peroxidases and manganese peroxidases which are key for the breakdown of lignin. As its genome sequence contains less genes predicted to encode cellulases, it grows less well on cellulose, which is typical for white-rot fungi. *F. fomentarius* was thus recently ranked with a moderate hyphal expansion rate on lignocellulosic substrates but a high rate of decomposition of its substrate when compared to another 20 basidiomycetes [22].

Another important premise for our decision was that *F. fomentarius* grows well on local agricultural residues such as hemp shives or rapeseed straw [16]. Hemp was once an important source for fibres for the textile industry, but its cultivation and use declined in the last century because cotton and synthetic fibres became more popular. The worldwide annual area of hemp harvested mainly for seeds and fibres is reported with about 150,000 ha for 2018 [23]. In Europe alone, the area under cultivation has increased to over 40,000 ha in recent years, which represents a potential use for at least 60,000 t of shives [23]. Hemp fibres currently experience a resurgence of interest by the textile industry as an environmentally friendly alternative to cotton, the cultivation of which is high in water demand, pesticide use and soil salinization [23]. In contrast, hemp is a frugal but high-yielding plant that has no pesticide and low fertilizer demand but uses water about six times more efficiently for biomass formation than cotton [24, 25]. Thus, hemp can grow well even under hot and dry conditions and on poor-soil sites such as prevalent in the Berlin-Brandenburg area and beyond [26]. The second main product after hemp fibre separation, the shives, are currently very often under-valued in applications like animal bedding. But with its content of about 48% w/w cellulose, 21 to 25% w/w hemicellulose and 17–19% w/w lignin [27], hemp shives are ideal substrates for both white-rot and brown-rot basidiomycetes.

Rapeseed will remain an important source of oil produced for food and feed as well as technical use, although it has a high water and fertilizer demand and the land use efficiency can be regarded as critical in terms of biodiesel production due to the low energy efficiency [28]. The Food and Agriculture Organization of the United Nations lists the harvested area of rapeseed as 36.96 Mio ha. In Brandenburg, winter rapeseed is the most important oilseed crop with an acreage of about 77,000 ha [29], which takes up about 10% of the arable land [30]. The composition of rapeseed straw is very similar to hemp shives with about 37% w/w cellulose, 24% w/w hemicellulose and about 17% w/w lignin [31] and thus well suited as a substrate for both white-rot and brown-rot basidiomycetes.

In the current study, we describe the cultivation of *F. fomentarius* on both hemp shives and rapeseed straw for the production of composite materials. We applied compression tests to determine the compressive Young’s Modulus as recently described for composite materials obtained with *Schizophyllum*, *Ganoderma* and *Trametes* species, respectively [7, 32, 33] and used scanning electron microscopy to characterize the composite structure and mechanical properties. We used the experimental data for numerical simulations of the compression behaviour. We furthermore studied the impact of the substrate’s particle sizes on the composite material properties and used fine, medium and coarse fractions of hemp shives and rapeseed straw to produce a set of diverse composite materials. Their properties were compared amongst each other and with the benchmark expanded polystyrene (EPS), a petroleum-based foam used for thermal isolation in the construction industry.

## Results and discussion

### Substrate preparation and classification

**Fig. 1:**
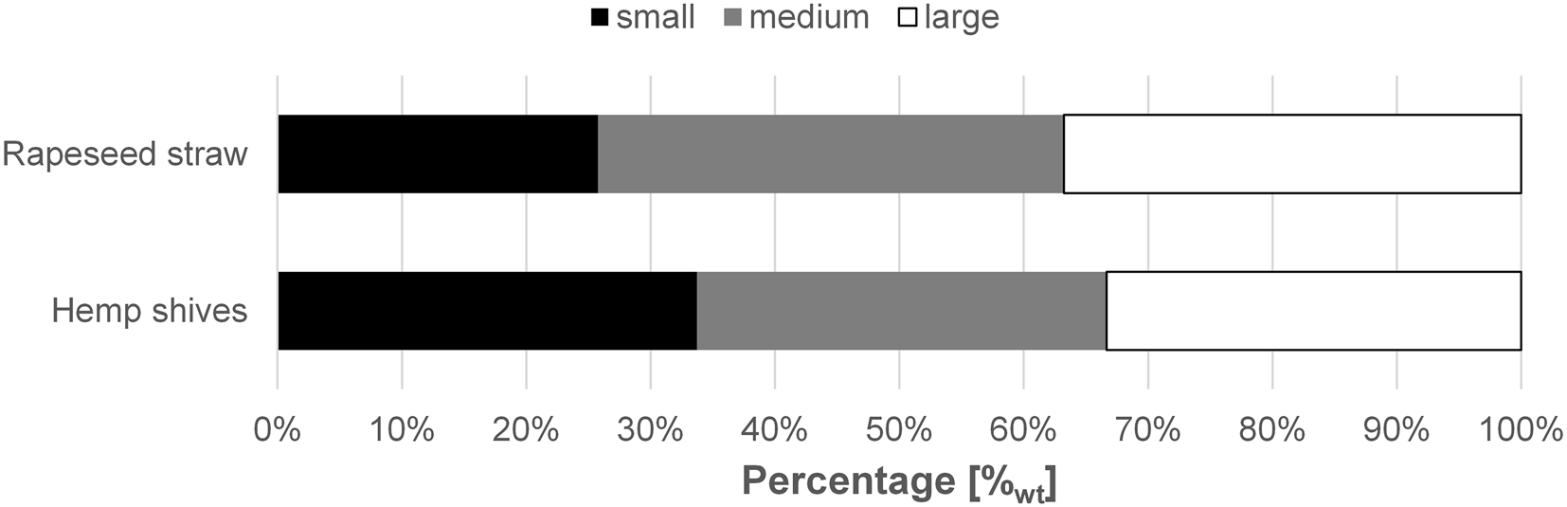
Mass distribution of plant substrate fractions after classification

The particle size of both hemp shives and rapeseed straw substrates were reduced by means of a laboratory cutting mill. To estimate the mass percentages of the subsequent classification products, sieve analyses of the milling products were carried out using analytical sieves and shakers. Rapeseed straw showed significantly larger amounts of screening residue of mesh sizes above 8 mm (Additional file 1). Furthermore, fine fractions below 0.63 mm mesh size were found at about 5% weight fraction. To achieve a mass distribution of approximately one third each for small, medium, and large fraction, the results suggested classification cut sizes of 2 mm and 3.15 mm, given the available screens. Consequently, these mesh sizes were utilized during the following classification processes via a Mogensen Sizer. Simultaneously, a 0.65 mm screen was used with the intention to reduce the finest particles such as dust. Hence, three particle fractions were prepared for each substrate: small (0.63–2 mm), medium (2–3.15 mm) and large (> 3.15 mm–6.3 mm). The resulting mass percentages are shown in Fig. 1.

**Fig. 2:**
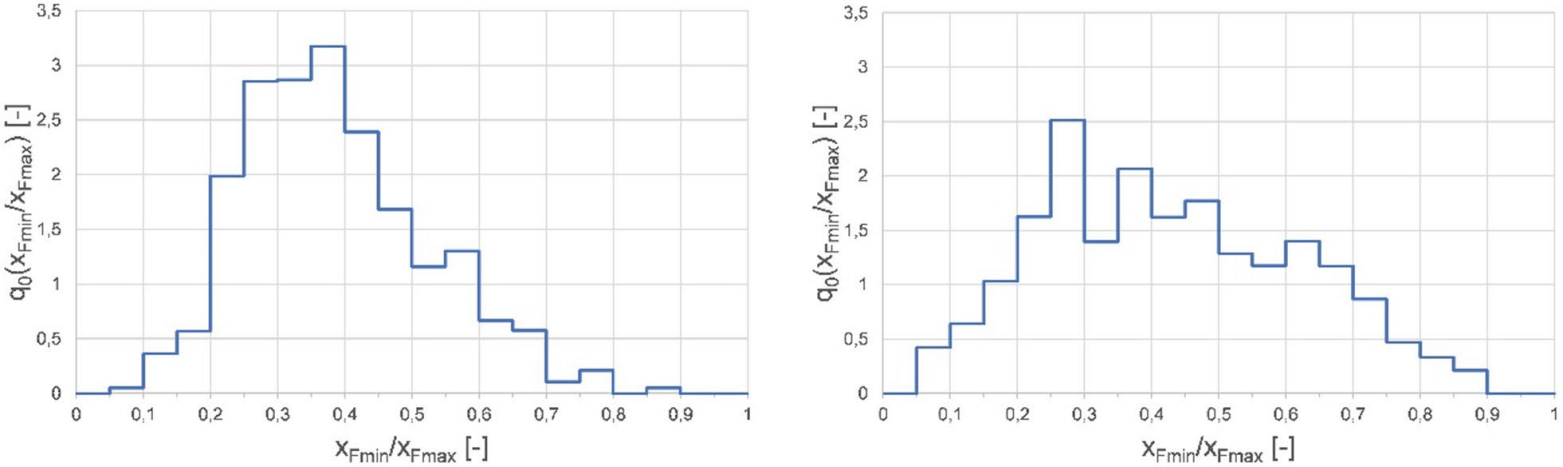
Histograms of minimum to maximum Feret diameter ratios for hemp shives (left) and rapeseed straw (right)

Finally, bulk density was determined for all fractions by measuring the total bulk volumes of the fractions (Additional file 2). Each fraction was subjected to further analysis with respect to particle size and shape by means of digital image analysis. A significantly larger number of finest particles were found within all rapeseed straw samples compared to hemp shives, possibly due to differences in abrasion resistance (note that results from image analysis are based on the number of particles rather than mass percentages). In Fig. 2, histograms of the ratios of minimum to maximum Feret diameters of hemp shives and rapeseed straw middle fractions are depicted. While the rapeseed straw’s modal value is smaller than the corresponding value for hemp shives, its distribution is broader and leans towards larger Feret ratios. Within the examined fraction, hemp shives display thinner and more elongated shapes.

### Cultivation of *F. fomentarius* and manufacturing of composite materials

**Fig. 3:**
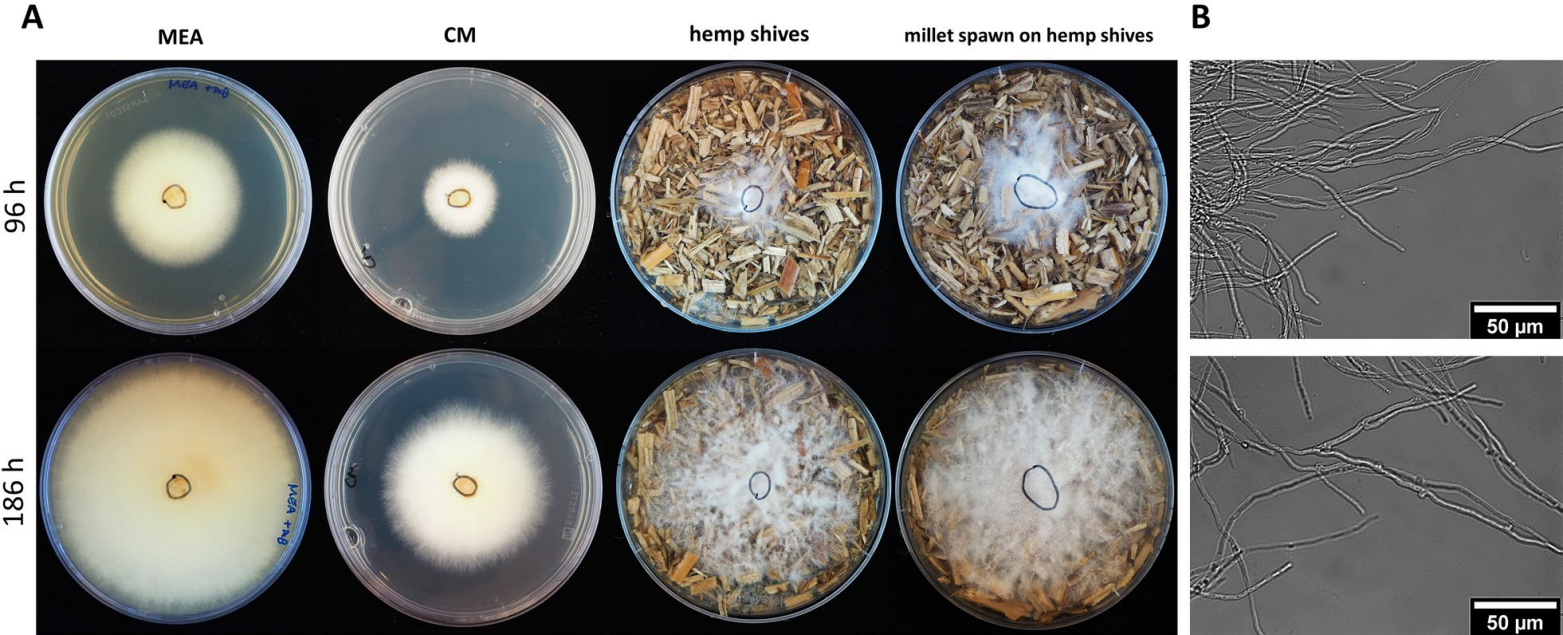
**A** *F. fomentarius* colonies after incubation at 25 °C in the dark for 96 h and 186 h. Doubling time of colony surface area on MEA and CM are 6 h (n = 8, SD = 1) and 14 h (n = 8, SD = 2) respectively. As the fungus also grows into the shives and towards the bottom of the agar plate, it is impossible to estimate a doubling time based on radial growth measurement when cultivated on hemp shives inoculated with pure *F. fomentarius* mycelium or with millet spawn. **B** Light microscopic images of *F. fomentarius* hyphae when cultivated in liquid CM for 96 h and 186 h, respectively at 400× magnification

**Fig. 4:**
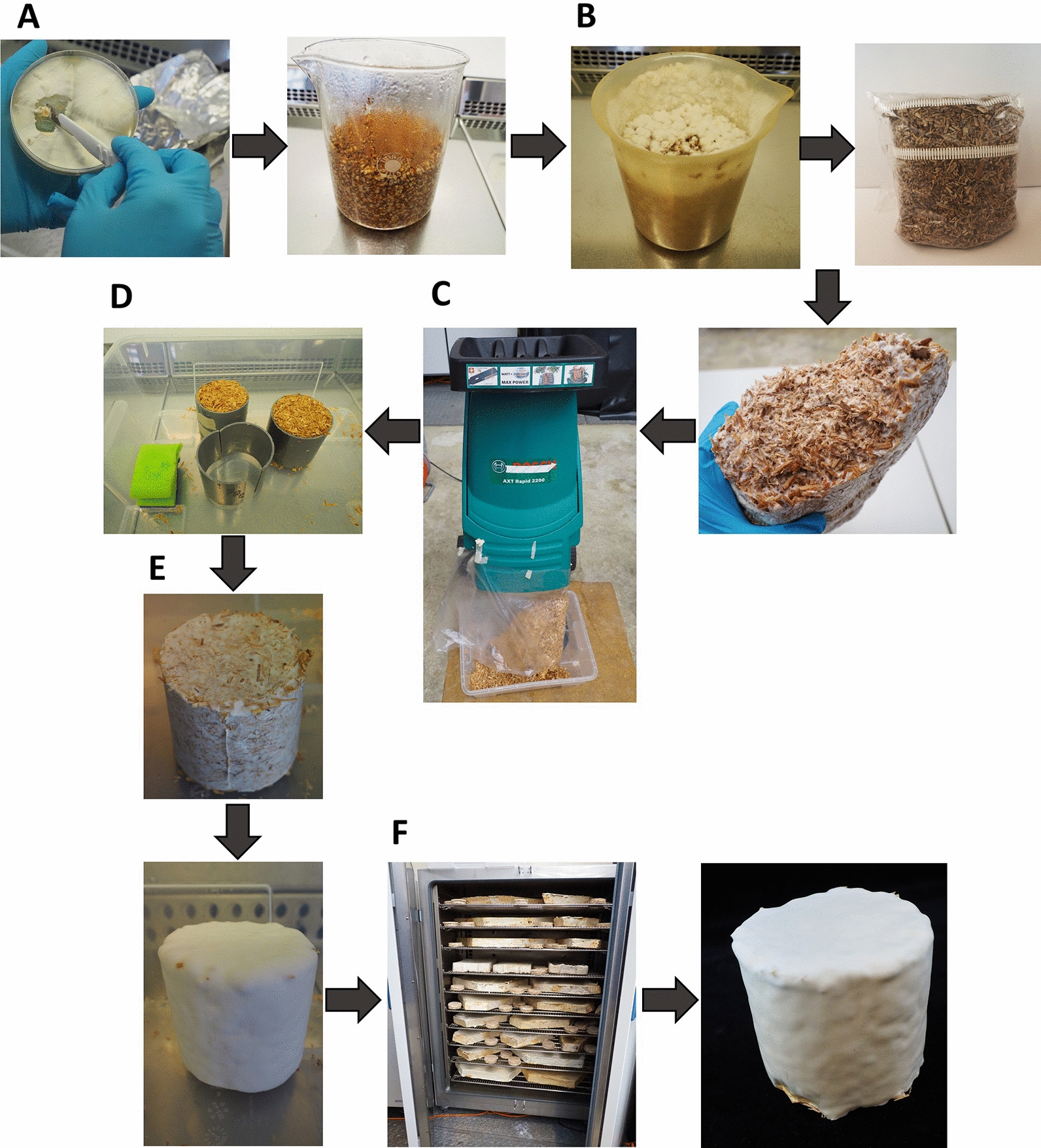
Laboratory manufacturing process for *F. fomentarius* composite materials. **A** Inoculation of sterile millet with for *F. fomentarius* mycelium followed by an incubation for 2 weeks at 25 °C in the dark. **B** Inoculation of hemp shives (or rapeseed straw) cultivation bags with the millet spawn followed by an incubation for 1 week at 25 °C in the dark. Note that the use of millet spawn for inoculation has the advantage of good mixing properties in the 3-L cultivation bags used and thus generation of more homogeneous growth throughout the plant substrates. **C** Shredding of preliminary hemp shives (or rapeseed straw) composites and transfer of the material into moulds. **D** Filled moulds before cultivation for 1 week at 25 °C in the dark. **E** Sample appearance after 1 week of cultivation. Moulds are removed to allow thorough overgrowth of the samples for another week. **F** Drying in an oven at 60 °C for 2 days and final appearance of composites used for compression tests

**Fig. 5:**
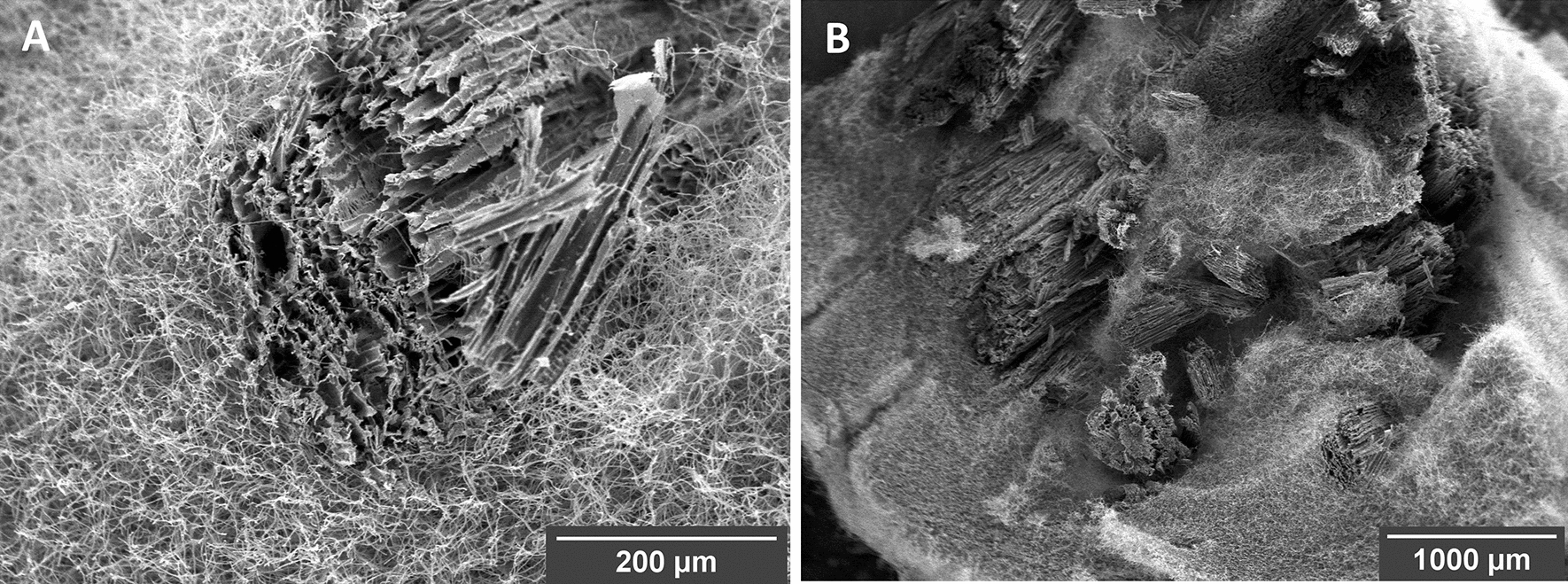
SEM images of *F. fomentarius* grown on hemp shives. **A** Overview of mycelium embedding a central cluster of hemp shives (centre); **B** Close up of hemp shives overgrown with mycelium, demonstrating that a dense mesh of mycelium connects the substrate particles. Sample specimens were taken from the outer zone of a composite body

*F. fomentarius* grows well on malt extract agar (MEA), glucose-based complete medium (CM) and on lignocellulosic substrates such as hemp shives and forms hyphae with a mean diameter of 2.8 µm (n = 300, SD = 0.7, Fig. 3). A three-stage laboratory manufacturing process was established for *F. fomentarius* (for details see “Methods” section). In the first stage, mycelium harvested from malt agar plates (Fig. 4A) was used to inoculate millet grains to obtain precultures of *F. fomentarius* during a 2-week cultivation (Fig. 4B). This ‘millet spawn’ then served as inoculum to inoculate 3-L bag cultures of hemp shives and rapeseed straw for the second stage cultivation. For future industrial upscaling efforts, however, we propose that the millet preculture should be substituted by non-food plant substrates that become mixed with non-inoculated plant substrates to avoid extensive use of cereal grains. After the 2-week cultivation in substrate bags, the overgrown substrates were shred and transferred into sterile cylindrical moulds (Fig. 4C and D), to allow for a final cultivation with the duration of 2 weeks, whereby the moulds were removed after one week (Fig. 4E). For each condition tested (substrate, particle fraction), at least six biological replicates were produced. The final composite materials obtained with this manufacturing process were optically inspected after cutting, revealing a gradient of fungal growth within the test specimens (Additional file 3). The outer shell of the material is covered by a dense pure mycelium of *F. fomentarius* and the inner part is less overgrown but still consists of sufficient hyphal material that covers and embeds all plant particles to keep them in place after cutting. Scanning electron microscopy (SEM) revealed that the composite material is formed by hyphae that form an isotropic network and interact with the hemp shives particles (Fig. 5). As for example described for *Trametes versicolor* and different types of agricultural feedstocks [33], these interactions eventually define the mechanical properties of the composite material at the microscale, something, which remains to be shown for *F. fomentarius* in future studies. On the macroscale, it appears that the outer layer is clearly beneficial to achieve a certain resistance against abrasion (Additional file 3).

### Compression tests of composites

Cylindrical composite specimens containing fine, medium and large particles of rapeseed straw or hemp shives where subjected to compression tests. At least six biological replicates per condition were used to determine their deformation behaviour. The results were compared with EPS. Hereby, the strain (the change in shape, that is the decrease in height divided by the original height of the specimen) depending on the stress (the force acting on a cylinder divided by the original cross-sectional area) was measured and expressed in stress–strain curves. Note that during elastic deformation, the relationship between stress and strain is linear and reversible and described by the elastic modulus. With further loading, above a yield point, a specimen becomes plastically deformed or exhibits cracks, resulting in permanent deformation even after unloading from the previously applied force.

**Fig. 6:**
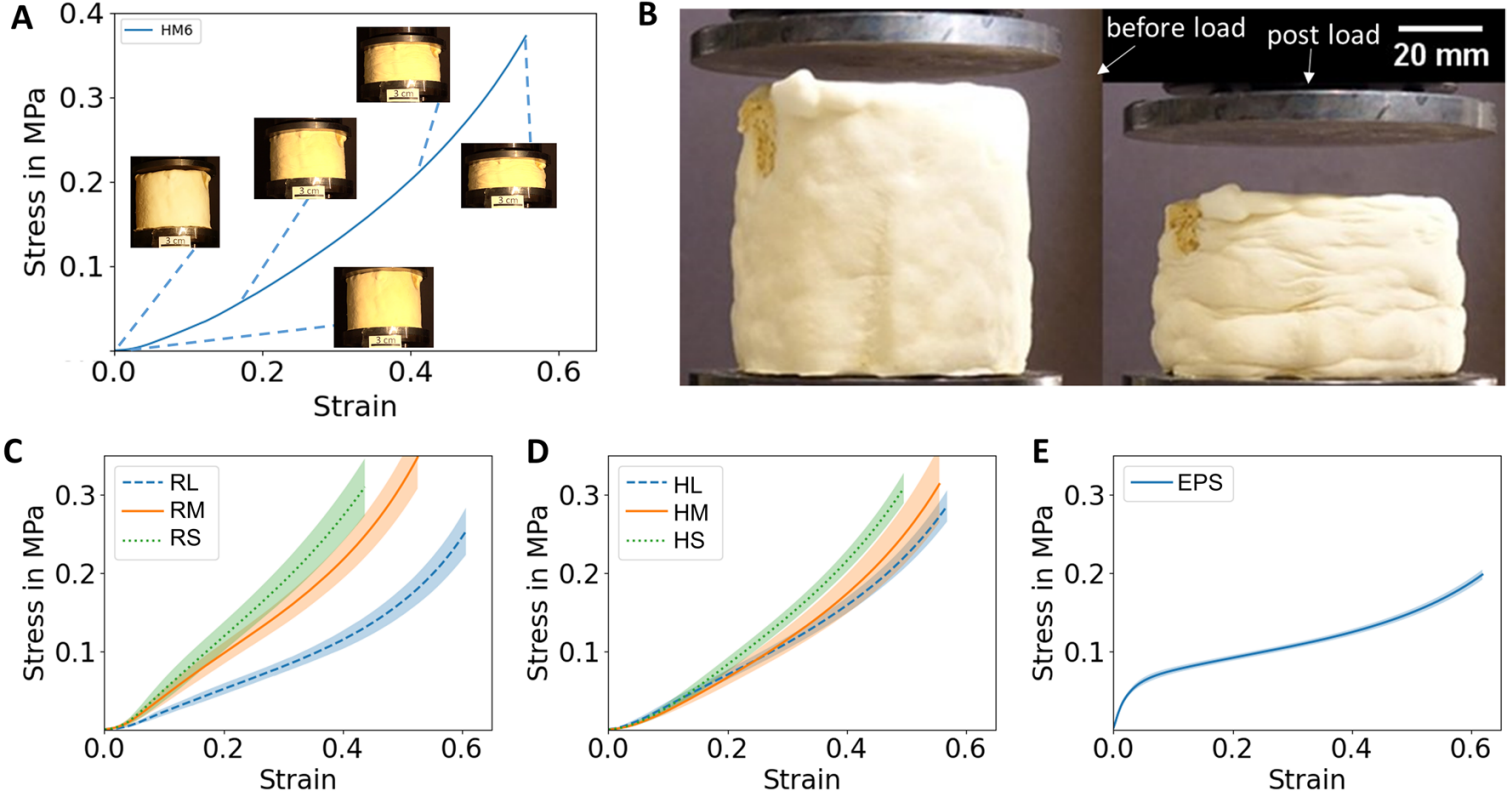
**A** Stress–strain curve of mycelium composite with medium sized substrate and optical micrographs highlighting the deformation at defined strain levels. **B** Compression test sample with medium size substrate particles before (left) and after (right) compression. **C**–**E** Compression stress–strain curves of (**C**) mycelium composite with rapeseed straw and (**D**) hemp shives of different particle sizes (RL, RM, RS—large, medium, small for rapeseed straw; HL, HM, HS—large, medium and small for hemp shives) and **E** EPS

During compression loading up to 1.8 kN (i.e. a force exerted by a weight of 180 kg), all mycelium composites showed elastic–plastic deformation behaviour. The stress–strain curves rise only slightly at the very beginning due to deformation of the surface mycelium on top of the composites. Following this region, the slope increases continuously until the end of the experiment. With increasing load, the specimens became clearly deformed (Fig. 6A, B). The elastic deformation recovered immediately after unloading from the compression tests, while a certain fraction of plastic deformation remained, which is depicted in Fig. 6B. While the specimens showed nearly no cracks and visible damage on the surface, we cannot exclude that cracks within the mycelium and/or delamination at the mycelium-reinforcement interfaces added to the remaining deformation.

Figures 6C–D compare the mean stress–strain curves with a confidence interval of 95% (shaded region) of composite material with different substrate material in the three different particle sizes large, medium and small. Notably, a significant difference can be seen between the individual particle sizes. Composites with large particle size performed less well in terms of compression stability compared to materials based on medium-sized particles. The composite materials with small particle performed the best, i.e. displayed the lowest elastic–plastic deformation for the same stress. Compared to EPS (Fig. 6E), however, the stress–strain relationships for the fungal composites scatter to a much greater extent, presumably due to inhomogeneous growth of *F. fomentarius* around and into the substrate particles and inhomogeneous substrate characteristics (see Fig. 5). However, when scaling fungal composite production from laboratory to industrial conditions, it will become possible to standardize substrate density, e.g. by applying precompression and weighing filled moulds, thus reducing biological variation. However, scatter as low as observed for purely synthetic material such as EPS will likely not be possible due to within- and between-subject variations which is an inherent property of biological systems.

**Fig. 7:**
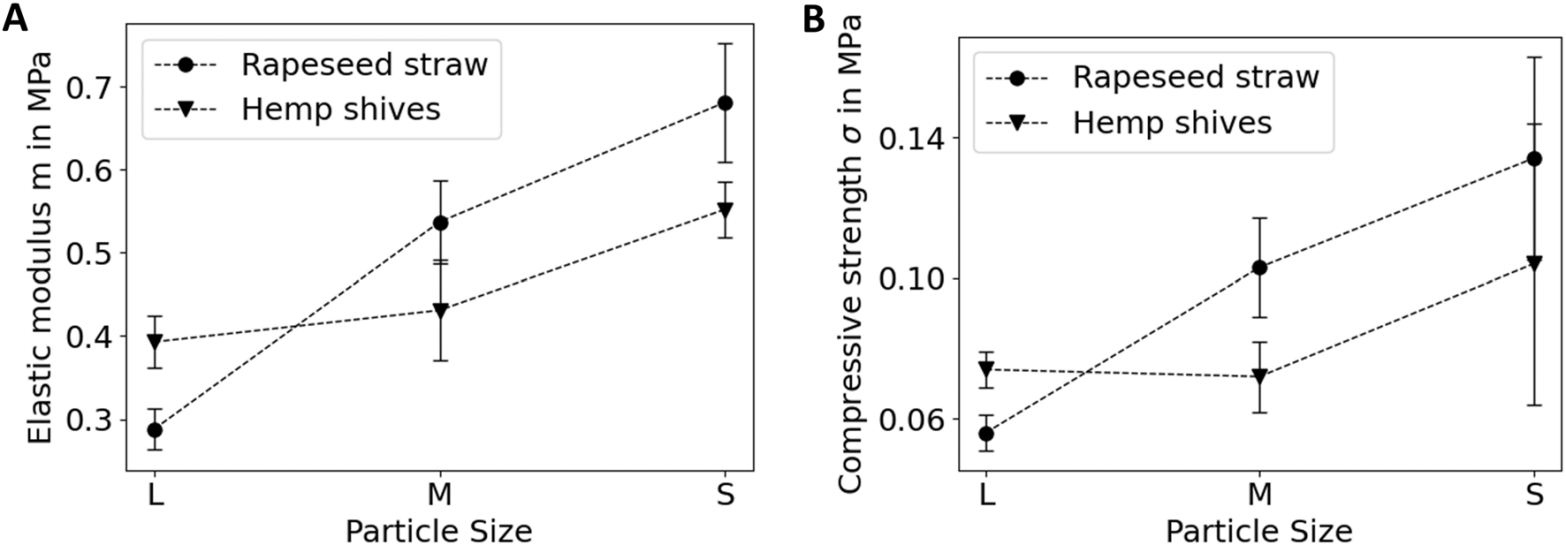
The elastic modulus m (**A**) and the compressive strength σ_st_ (**B**) dependent on the particle sizes large (L), medium (M) and small (S) of rapeseed straw and hemp shives, respectively. For additional data, see Additional file 5

For calculation of the elastic modulus m, we decided to evaluate the range of the curve, where the cross-section of the samples was equally loaded, namely from 10% strain (unequal loading is due to the inclination of some samples) up to a strain value estimated by the elastic recovery. The slope of this part of the curve corresponds to the elastic modulus. Furthermore, the compressive strength σ_st_ was evaluated at 20% strain. Figure 7 and Additional file 4 compare the elastic modulus and the compression strength for the composites based on different substrate particle sizes. Remarkably, as compared to composites with hemp shives, composites with rapeseed straw particles performed slightly better in the medium and small size range (compressive strength of 0.103 ± 0.014 MPa for medium and 0.134 ± 0.029 MPa for small size compared to 0.072 ± 0.010 MPa and 0.104 ± 0.040 MPa for medium and small hemp shives). In contrast, composites based on large hemp shive particles performed better compared to those with large rapeseed straw particles (compressive strength of 0.056 ± 0.005 MPa for rapeseed straw, 0.072 ± 0.005 MPa for hemp shives). Interestingly, the influence of particle size on the elastic moduli is the same as for the strength: large hemp shive particles lead to a higher modulus than large rapeseed straw particles, whereas medium and small rapeseed straw particles lead to higher modulus values as compared to hemp shive particles in that size range.

The stress–strain behaviour of EPS is significantly different from the mycelium composites. It shows an elastic modulus of 2.37 ± 0.17 MPa, which is 4-times higher compared to the *F. fomentarius* composite materials, but it has a compressive strength of 0.09 ± 0.003 MPa, which is in the range of the fungal composite material. However, if the load bearing capability at higher strains, e.g. 50% is compared, the composites exhibit stress values comparable to EPS.

### Numerical simulations of the composite material behaviour

Numerical simulations of the compression tests on the composite materials were performed using the finite element method (FEM) [34]. One practical approach is to consider the specimens as homogenous isotropic solids. However, given the large range of strains exhibited by the specimens in the compression tests before plastic deformation, the usage of a linear strain tensor in a model is erroneous. Thus, fully non-linear kinematics must be applied and a compressible variant of the hyperelastic Neo-Hookean model [35] was chosen as the constitutive model (see “Methods” section).

**Fig. 8.**
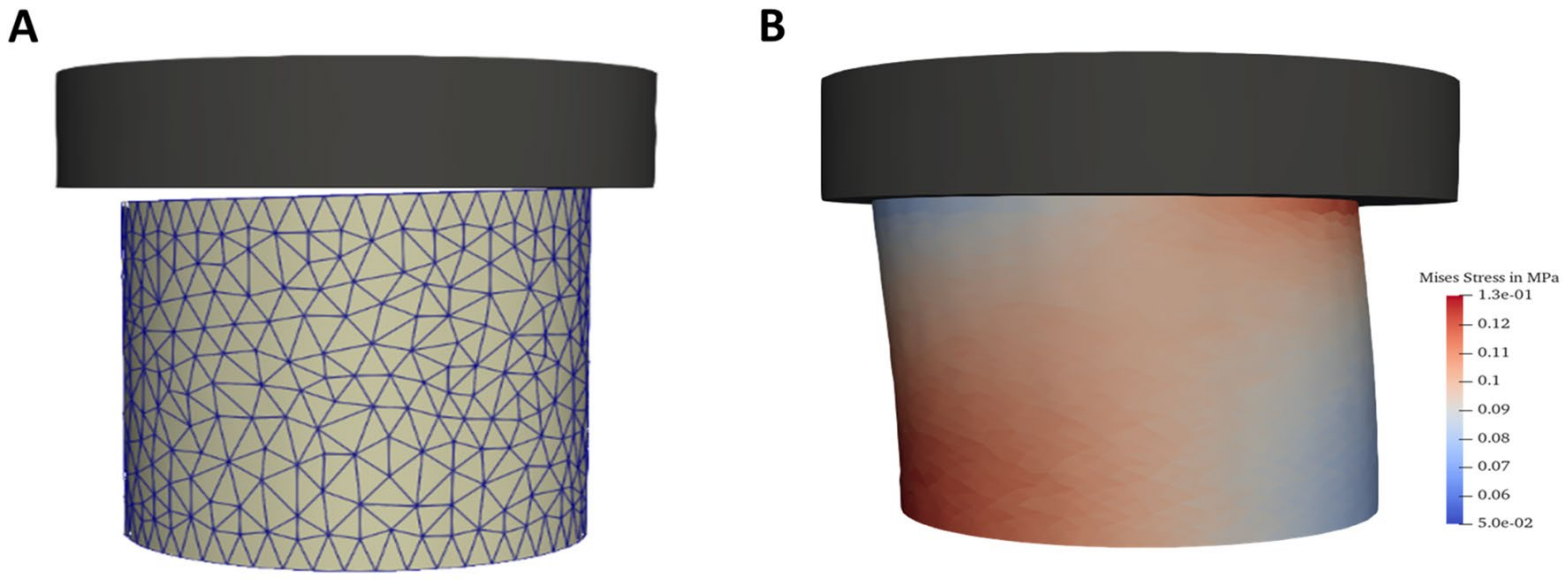
3D FEM simulation of compression tests. **A** Slanted mesh used in simulations to reflect the initial. **B** Deformed configuration due to contact pressure, colouring represents equivalent stress in the specimen. For details regarding numerical simulations see “Methods”

**Fig. 9:**
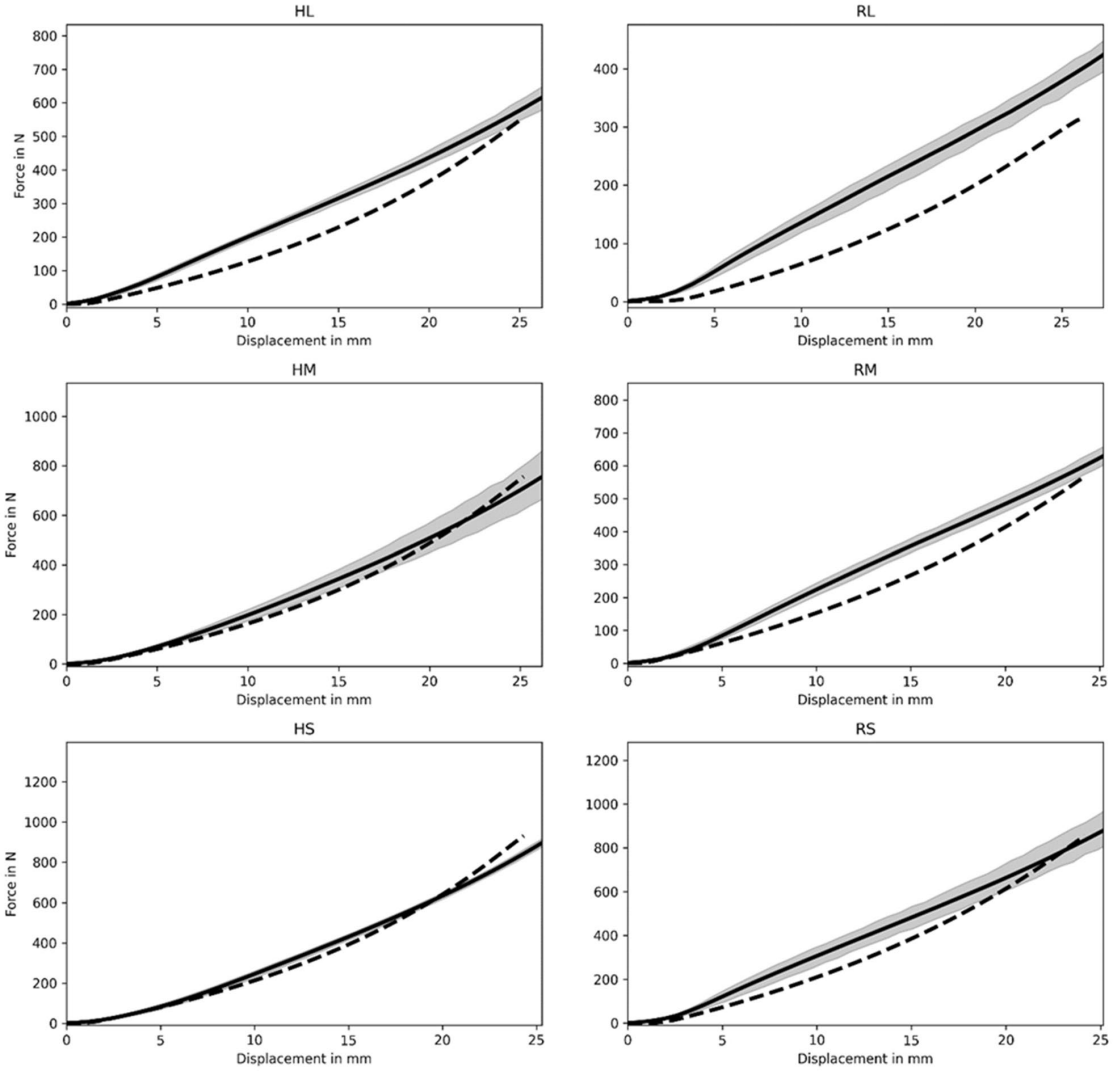
Force–displacement data as computed using FEM with the Neo-Hookean model (dashed line) and experimental data (straight line). Particle sizes are RL, RM, RS—large, medium, small for rapeseed straw; HL, HM, HS—large, medium and small for hemp shives

An example 3D visualization before a simulated compression test is shown in Fig. 8 and the force–displacement simulation results are shown in Fig. 9. Although the model reflects the qualitative nonlinear behaviour well, quantitative discrepancies can be identified. These are expected and can be traced back to various reasons. Most importantly, some important mechanical effects occurring in the composite material are not reflected in the homogenous Neo-Hookean model. This includes damage as well as substrate debonding, but also surface effects (see Additional file 6) and differences between real specimen geometry and idealized cylindrical mesh geometries. Furthermore, although slanted geometries have been taken into account, the initial phase of the compression test where the specimen settles and full contact between stamp and specimen surface is established is not sufficiently well reflected in the simulations. This can be seen best in the data obtained for large rapeseed straw particles (RL, Fig. 9). A better representation of the specimen geometry could thus improve the simulation.

Remedy to the mechanical flaws of the model can either be provided by explicitly simulating the composite including damage and debonding or using a dedicated homogenous model specially developed for the novel material combination at hand. Nevertheless, based on the data the model used provides a good qualitative reflection of the composite’s behaviour in compression.

## Conclusions

In this study, we investigated the impact of rapeseed straw and hemp shives particle sizes on the characteristics and compression behaviour of mycelium composite materials produced by the basidiomycete *F. fomentarius*.

In general, the characteristics of mycelium composites are defined on multiscale levels. On the microscale level (µm), the smallest mechanically effective component of the material is the fungal network of hyphae that form the matrix, and whose elastic properties are mainly dependent on the cell wall chitin content [8]. On the mesoscale level (mm) are reinforcing substrate particles such as hemp shives and rapeseed straw that become bonded randomly by the mycelial network. Bonding in this heterogeneous material is discontinuous at the particle–matrix interface. The macroscale level is defined by the size of the final mycelium composites and can be measured by stress–strain curves. Our first simulation analyses, based on experimentally obtained compression data, cannot reflect yet the mechanisms occurring at the microscale and mesoscale and further experimental investigations are thus necessary. However, the chosen two-parameter model can qualitatively reflect the global force displacement behaviour and thus serve as a base model which can be augmented when additional experimental data is available to achieve a better quantitative agreement. To this end, a micromechanically informed damage model may be included to reflect the decreasing stiffness observed in some of the specimens. The different particle sizes are represented by significantly differing values for the constitutive parameters of the hyperelastic model chosen for the FEM simulations.

Notably, the impact of particle size on compression behaviour was more profound for large rapeseed straw particles, whereas stress–strain curves of other combinations of particle sizes and substrates appeared more clustered. In addition, the stress–strain curves of mycelium composites differed from stress–strain curves obtained for EPS. On the one hand, less force is required for initial deformation of the mycelium surface of the composites, showing that surface damage of the material occurs more easily than with EPS. However, when comparing the ability to take up compressive forces, both hemp shive-based and rapeseed-based composites take up more load compared to EPS before being deformed. At 50% compression, hemp-shive-based and rapeseed-based composites were able to resist compressive forces of 0.2 MPa and 0.3 MPa, respectively, whereas EPS only sustained 0.15 MPa. Thus, the composite materials obtained with *F. fomentarius* are in the same range or slightly above of the values obtained for EPS. The data obtained in this study thus suggest that *F. fomentarius* potentially constitutes a promising cell factory for the future development of fungal composite materials that could replace synthetic foams such as EPS. However, other important material properties such as thermal insulation, water resistance, long-term stability, aging, biodegradability to name but a few need to be studied and likely optimized in future experiments. The advantage of composite materials based on *F. fomentarius* over synthetic ones is that their characteristics can likely be easily modulated at the nano- and micro-scale through variations of the cultivation conditions of *F. fomentarius* and thus its cell wall composition [36].

## Methods

### Substrate preparation and classification

Two types of lignocellulosic substrate were tested: hemp shives (Hemparade) and rapeseed straw (Optistraw) from European agriculture, both purchased from Futtermittel Louven e.K. For each substrate, three particle fractions were prepared: small (0.63–2 mm), medium (2–3.15 mm) and large (> 3.15 mm–6.3 mm). Processing was tailored to the specific requirements of the substrate type. Preliminary size reduction was optionally carried out by means of a laboratory cutting mill with an 8 mm discharge screen, constraining the particle size distribution if necessary. To achieve fractionation, each substrate was classified by using a three-deck Mogensen sizer with mesh sizes of 0.63 mm, 2 mm, and 3.15 mm. The densities of the resulting fractions were determined by measuring their total masses and bulk volumes. The fractions were finally subjected to detailed analysis of particle size and shape. The images were taken via a flatbed scanner and digital microscopy; Zeiss Zen software was employed for image analysis.

### Isolation and cultivation of *F. fomentarius*

The *F. fomentarius* isolate GaG41 is particularly suitable to produce composite materials from agricultural raw materials, as described previously [16]. As growth performance of fungi is often strain-dependent, we isolated additional *F. fomentarius* strains form the Berlin-Brandenburg area (Germany). One of them, strain PaPF11, showed better growth rate on malt extract agar, glucose agar medium, millet culture (used for millet spawn production) and solid lignocellulosic substrates such as hemp shives and rapeseed straw compared to GaG41 (data not shown) and was thus used in the current study.

In brief, strain PaPF11 was isolated using a fruiting body collected from a birch tree trunk. Isolation was performed by cutting slants (3 × 3 mm) from different internal zones of the fruiting body, followed by dipping into 4% H_2_O_2_ solution for 30 s to reduce bacterial burden. Slants were placed on malt extract agar plates (Roth, Germany) supplemented with 50 µg/ml ampicillin sodium salt (Sigma-Aldrich) and 50 µg/ml streptomycin sulfate (Applichem) to suppress bacterial growth. Mycelium outgrown from the slants was transferred twice to new plates using sterile toothpicks to obtain axenic cultures. Strain identity was confirmed by Sanger sequencing of the internally transcribed spacer (ITS) region using primer ITS1 (TCCGTAGGTGAACCTGCGG) and ITS4 (TCCTCCGCTTATTGATATGC) as described earlier [37]. For strain maintenance, cultures were grown for about 2 weeks in the dark at 25–27 °C followed by cold storage at 2–8 °C and subsequent transfer of mycelium pieces to new medium plates. The same cultivation conditions were also used for preparation of mycelium plates for millet spawn inoculation.

### Manufacturing of composite materials

Strain PaPF11 was harvested from an agar plate after 5–7 days of cultivation and used to inoculate a brown millet culture (purchased from Mühle Schlingemann, Germany), which served as preculture to inoculate the bulk solid substrate. Brown millet was supplemented with 1 wt.% calcium sulfate dihydrate (Roth) and 150 wt.% distilled water. The mixture was sterilized by autoclaving (VX-150 autoclave, Systec GmbH, Germany) and incubated for 14 days at 25 °C in the dark after inoculation.

Particle fractions from hemp shives and rapeseeds straw were hydrated with 150 wt% of water in separate cultivation bags (SacO2, Belgium) and autoclaved. 5 wt% overgrown millet spawn was added to the wet substrate and mixed by kneading. The bags were then heat sealed and incubated at 25 °C in the dark. After 7 days of incubation, the bags were mixed to promote homogeneous growth and incubation was continued for another 7 days. The overgrown solid substrate was then crushed using a disinfected shredder (Rapid AXT 2000, Bosch, Germany) and manually transferred into a plastic tube of 7 cm diameter and 6–7 cm height which served as a cylindrical mould. Note that it was impossible to control exactly the amount of pre-compression of the crushed mycelium-substrate mix to the tube. Therefore, for each condition tested (substrate, particle fraction), six biological replicates were produced. The samples were incubated for 7 days in the mould followed by another 7 days after removing the mould to allow surface growth of *F. fomentarius*. To reduce the risk of contamination and ensure a high relative humidity of 80–100%, incubation was done in a disinfected closed plastic box (IKEA, Sweden) with two sterile sponges soaked in sterile distilled water. Finally, growth of *F. fomentarius* was stopped by drying the samples in an oven (B5090E, Heraeus, Germany) at 60 °C for 2 days. The weight and geometry parameters of the produced samples were recorded, and the density of the specimen calculated to access the reproducibility of this manufacturing process (data not shown). As a reference for expanded polystyrene (EPS), commercially available EPS plates (FIW, Germany) with a thermal conductivity coefficient of 0.035 W/mK according to DIN 4102-1: B1 where cut to the same geometry as fungal samples using a scalpel.

### Microstructural characterisation

Scanning electron microscopy (SEM, CamScan Series 2, Obducat, Sweden) was performed to analyse hyphal growth of *F. fomentarius* on lignocellulosic substrates. In brief, SEM was used in the high vacuum, secondary electron mode with an accelerating voltage of 14 kV. The specimen was gold sputtered (Cressington Sputter Coater, 108 Auto, Tescan GmbH, Dortmund, Germany) for 40 s at 30 mA.

### Compression testing

Cylindrical specimens (at least six biological replicates for each substrate/particle fraction combination) underwent compression testing in a universal testing machine type 0008.00 (UTS Testsysteme GmbH, Ulm, Germany) with a crosshead speed of 10 mm/min and a pre-load of 1 N. Load and displacement were measured by a 2 kN load cell (resolution 0.01%) and the in-built displacement transducer (resolution 0.001%). The dimensions of each specimen were measured with callipers to calculate the stress σ and the strain ε (note that stretch in material science is called ‘strain’ which is different from the meaning of the term ‘strain’ in microbiology). The tests ended automatically at a load of 1.8 kN or earlier when a certain displacement (RS 32 mm, RM 42 mm, RL 44 mm, HS 30 mm, HM 35, HL 40) was reached. Immediately after the test, the height of the samples was determined.

The stress–strain curves were evaluated according to the German standard for compression testing of foams DIN 50134:2008-10 [38]. The load–displacement curves were converted to stress–strain curves, using the following formulas to calculate the stress σ and the strain ε: $\sigma = \frac{F}{A}\left[ {{\text{MPa}}} \right]$ and $\varepsilon = { }\Delta L/L_{0} \left[ - \right]$ where F equals the compressive force [N], A is the original cross-sectional area of the specimen [mm^2^], ΔL is the obtained displacement [mm] and L_o_ corresponds to the original height of the specimen [mm].

### Numerical simulations

A compressible variant of the hyperelastic Neo-Hookean model [35] was chosen as the constitutive model to describe the characteristics of the composite materials. The associated hyperelastic potential, i.e. the strain energy density, reads

$$w\left( {{\uplambda }_{i} } \right) = c\left( {{\uplambda }_{1}^{2} + {\uplambda }_{2}^{2} + {\uplambda }_{3}^{2} } \right) - 3 - 2\ln \left( {\lambda_{1} \lambda_{2} \lambda_{3} } \right) + d\left( {\lambda_{1} \lambda_{2} \lambda_{3} - 1} \right)^{2} .$$

Here, $\lambda_{i}$ are the principal stretches, and $c$ and $d$ are material parameters which need to be identified using experimental data. For the parameter identification process based on the 1D experimental data at hand a uniaxial simplification of the model is needed. The second Piola–Kirchhoff stress tensor ${\varvec{S}}$ is then given as derivative of the potential which in the present case of uniaxial compression can be simplified to

$${\varvec{S}} = 2\frac{\partial w}{{\partial {\varvec{C}}}} = 2\mathop \sum \limits_{i} \frac{{\partial w\left( {\lambda_{k} } \right)}}{{\partial \lambda_{i} }}\frac{{\partial \lambda_{i} }}{{\partial {\varvec{C}}}} = \mathop \sum \limits_{i} \frac{1}{{\lambda_{i} }}\frac{\partial w}{{\partial \lambda_{i} }}{\varvec{e}}_{i} \otimes {\varvec{e}}_{i} .$$

The requirement that the specimen be stress free in directions perpendicular to the loading direction (${\varvec{e}}_{3}$) leads to a closed form expression for the uniaxial stress $S_{33}$ as a function of the stretch $\lambda = \lambda_{3}$ in loading direction:

$$S_{33} \left( \lambda \right) = 2c\left( {1 - \frac{1}{{\lambda^{2} }}} \right) + d\frac{\psi \left( \lambda \right)}{{\lambda^{3} }} \left( {\frac{1}{2\lambda }\psi \left( \lambda \right) - 1} \right) ,$$

$$\psi \left( \lambda \right) = \lambda - \frac{c}{d} + \sqrt {4\frac{c}{d}\lambda^{2} + \left( {\lambda + \frac{c}{d}} \right)^{2} } .$$

The above expression for the uniaxial stress is used to fit the model to the average experimental stress strain curve across specimens of identical substrate material and particle sizes. Using this approach, the influence of substrate type and particle size is entirely reflected in the values of the two model parameters.

With the constitutive parameters $c$ and $d$ at hand (Additional files 5 and 6) the compression tests are simulated using the open source finite element computing platform FeniCS [39]. A penalty contact algorithm was implemented together with slightly slanted top surfaces of the specimen meshes according to measured geometry data to model the compression boundary conditions.

## Supplementary Information

**Additional file 1: **Particle size distribution.

**Additional file 2: **Bulk densities of substrate fractions.

**Additional file 3: **Cut section of a composite.

**Additional file 4: **Elastic modulus and compression strengths of composites.

**Additional file 5: **Neo-Hookean material parameters.

**Additional file 6: **Comparison of the Neo-Hookean model data with experimental data.

## Data Availability

The raw datasets generated in this study are available from the corresponding authors upon request.

## References

[1] Meyer V, Andersen MR, Brakhage AA, Braus GH, Caddick MX, Cairns TC, de Vries RP, Haarmann T, Hansen K, Hertz-Fowler C (2016). Current challenges of research on filamentous fungi in relation to human welfare and a sustainable bio-economy: a white paper. Fungal Biol Biotechnol.

[2] Meyer V, Basenko EY, Benz JP, Braus GH, Caddick MX, Csukai M, De Vries RP, Endy D, Frisvad JC, Cimerman NG (2020). Growing a circular economy with fungal biotechnology : a white paper. Fungal Biol Biotechnol..

[3] Gandia A, van den Brandhof JG, Appels FVW, Jones MP (2021). Flexible fungal materials: shaping the future. Trends Biotechnol.

[4] Cerimi K, Akkaya KC, Pohl C, Schmidt B, Neubauer P (2019). Fungi as source for new bio-based materials: a patent review. Fungal Biol Biotechnol.

[5] Yang L, Park D, Qin Z (2021). Material function of mycelium-based bio-composite: a review. Front Mater.

[6] Appels FVW, van den Brandhof JG, Dijksterhuis J, de Kort GW, Wösten HAB (2020). Fungal mycelium classified in different material families based on glycerol treatment. Commun Biol..

[7] Appels FVW, Camere S, Montalti M, Karana E, Jansen KMB, Dijksterhuis J, Krijgsheld P, Wösten HAB (2019). Fabrication factors influencing mechanical, moisture- and water-related properties of mycelium-based composites. Mater Des.

[8] Islam MR, Tudryn G, Bucinell R, Schadler L, Picu RC (2017). Morphology and mechanics of fungal mycelium. Sci Rep.

[9] Jones M, Chun H, Yuen R, John S (2018). Waste—derived low—cost mycelium composite construction materials with improved fire safety. Fire Mater.

[10] Elsacker E, Vandelook S, Damsin B, Van Wylick A, Peeters E, De Laet L (2021). Mechanical characteristics of bacterial cellulose-reinforced mycelium composite materials. Fungal Biol Biotechnol.

[11] Vandelook S, Elsacker E, Van Wylick A, De Laet L, Peeters E (2021). Current state and future prospects of pure mycelium materials. Fungal Biol Biotechnol.

[12] Chen H, Abdullayev A, Bekheet MF, Schmidt B, Regler I, Pohl C, Vakifahmetoglu C, Czasny M, Kamm PH, Meyer V (2021). Extrusion-based additive manufacturing of fungal-based composite materials using the tinder fungus *Fomes Fomentarius*. Fungal Biol Biotechnol.

[13] Elsacker E, Vandelook S, Van Wylick A, Ruytinx J, De Laet L, Peeters E (2020). A comprehensive framework for the production of mycelium-based lignocellulosic composites. Sci Total Environ.

[14] Tacer-Caba Z, Varis JJ, Lankinen P, Mikkonen KS (2020). Comparison of novel fungal mycelia strains and sustainable growth substrates to produce humidity-resistant biocomposites. Mater Des.

[15] Jones M, Gandia A, John S, Bismarck A (2021). Leather-like material biofabrication using fungi. Nat Sustain.

[16] Meyer V, Schmidt B, Pohl C, Cerimi K, Schubert B, Weber B, Neubauer P, Junne S, Zakeri Z, Rapp R, et al. Mind the Fungi. In: Meyer V, Rapp R, eds. Universitätsverlag der TU Berlin: Berlin, 2020. 10.14279/depositonce-10350.

[17] Grienke U, Zöll M, Peintner U, Rollinger JM (2014). European medicinal polypores—modern view on traditional uses. J Ethnopharmacol.

[18] D`Aguanno MN, Dresch P, Grienke U, Rollinger JM, Peintner U, Rosam K (2015). Fungal strain matters: colony growth and bioactivity of the european medicinal polypores *Fomes Fomentarius, Fomitopsis Pinicola* and *Piptoporus Betulinus*. AMB Express.

[19] Papp N, Rudolf K, Bencsik T, Czégényi D (2017). Ethnomycological use of *Fomes Fomentarius* (L.) Fr. and *Piptoporus Betulinus* (Bull.) *P. Karst* in Transylvania, Romania. Genet Resour Crop Evol.

[20] Müller C, Klemm S, Fleck C (2021). Bracket fungi, natural lightweight construction materials: hierarchical microstructure and compressive behavior of *Fomes Fomentarius* fruit bodies. Appl Phys A.

[21] Hage H, Miyauchi S, Virágh M, Drula E, Min B, Chaduli D, Navarro D, Favel A, Norest M, Lesage-Meessen L (2021). Gene family expansions and transcriptome signatures uncover fungal adaptations to wood decay. Environ Microbiol.

[22] Lustenhouwer N, Maynard DS, Bradford MA, Lindner DL, Oberle B, Zanne AE, Crowther TW (2020). A trait-based understanding of wood decomposition by fungi. Proc Natl Acad Sci U S A.

[23] Carus M, Barth de MNB. Carbon footprint and sustain- ability of different natural fibres for biocomposites and insulation material; nova-Institute, Hürth, Germany, 2019. www.bio-based.eu/ecology. Accessed 27 Sept 2021.

[24] Drastig K, Flemming I, Gusovius H-J, Herppich WB (2020). Study of water productivity of industrial hemp under hot and dry conditions in Brandenburg (Germany) in the Year 2018. Water.

[25] Tang K, Struik PC, Yin X, Calzolari D, Musio S, Thouminot C, Bjelková M, Stramkale V, Magagnini G, Amaducci S (2017). A comprehensive study of planting density and nitrogen fertilization effect on dual-purpose hemp (*Cannabis Sativa *L.) cultivation. Ind Crops Prod.

[26] Herppich WB, Gusovius HJ, Flemming I, Drastig K (2020). Effects of drought and heat on photosynthetic performance, water use and yield of two selected fiber hemp cultivars at a poor-soil site in Brandenburg (Germany). Agronomy.

[27] Thomsen AB, Rasmussen S, Bohn V, Nielsen KV, Thygesen A (2005). Hemp raw materials: the effect of cultivar, growth conditions and pretreatment on the chemical composition of the fibres. Risø-R Rep.

[28] van Duren I, Voinov A, Arodudu O, Firrisa MT (2015). Where to produce rapeseed biodiesel and why? Mapping european rapeseed energy efficiency. Renew Energy.

[29] G. Barthelmes; Ebel, G. Sortenratgeber—2020/2021. Winterraps. https://www.isip.de/isip/servlet/resource/blob/265960/01f97d3a9bdb29aa67576bbceb2c388c/sr-w. Accessed 21 Sept 2021.

[30] Barthelmes G, Fahlenberg E, Zimmer J, Heidecke D, Hanff H, Schröder G, Radtke J, Adam L, Roschke M. Raps—Empfehlungen Zum Anbau in Brandenburg. 2009. https://www.brandenburg.de/sixcms/media.php/4055/raps.pdf. Accessed 21 Sept 2021.

[31] Díaz MJ, Cara C, Ruiz E, Romero I, Moya M, Castro E (2010). Hydrothermal pre-treatment of rapeseed straw. Bioresour Technol.

[32] Islam MR, Tudryn G, Bucinell R, Schadler L, Picu RC (2018). Mechanical behavior of mycelium-based particulate composites. J Mater Sci.

[33] Elsacker E, Vandelook S, Brancart J, Peeters E, De Laet L (2019). Mechanical, physical and chemical characterisation of mycelium-based composites with different types of lignocellulosic substrates. PLoS ONE.

[34] Zienkiewicz OC, Taylor RL, Zhu JZ. The finite element method: its basis and fundamentals. Elsevier; 2013. 10.1016/C2009-0-24909-9.

[35] Pence TJ, Gou K (2015). On compressible versions of the incompressible neo-hookean material. Math Mech Solids.

[36] Henning L, Simon U, Abdullayev A, Schmidt B, Pohl C, Nunez Guitar T, Vakifahmetoglu C, Meyer V, Bekheet MF, Gurlo A (2022). Effect of *Fomes Fomentarius* cultivation conditions on its adsorption 2 performance for anionic and cationic dyes. ACS Omega.

[37] White TJ, Bruns T, Lee S, Taylor J. Amplification and direct sequencing of fungal ribosomal rna genes for phylogenetics. PCR Protocols. Elsevier, 1990; p. 315–322. 10.1016/B978-0-12-372180-8.50042-1.

[38] DIN 50134:2008–10. Testing of metallic materials—compression test of metallic cellular materials. 10.31030/1443205.

[39] Alnæs MS, Blechta J, Hake J, Johansson A, Kehlet B, Logg A, Richardson C, Ring J, Rognes ME, Wells GN (2015). The FEniCS project version 1.5. Arch Numer Softw.

