# Supplementary figures and images for: Establishment of the basidiomycete Fomes fomentarius for the production of composite materials
Source: Fungal Biol Biotechnol. 2022 Feb 24;9:4. doi: 10.1186/s40694-022-00133-y (PMC8876124; doi:10.1186/s40694-022-00133-y)

## Slide 1
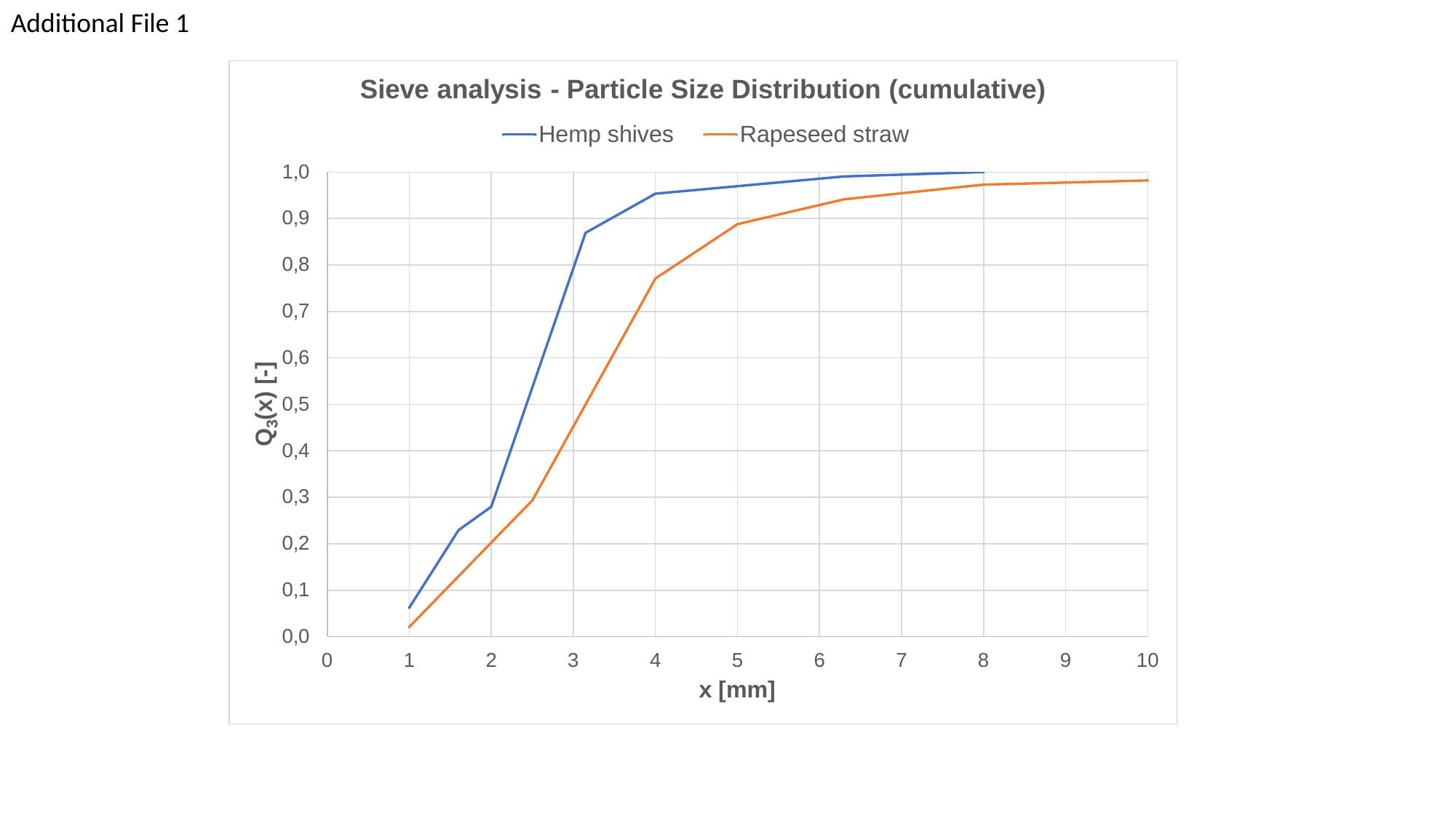

Additional File 1

Supplement: Supplementary file 1 — Additional file 1: Particle size distribution. [file 40694_2022_133_MOESM1_ESM.pptx]

## Slide 1
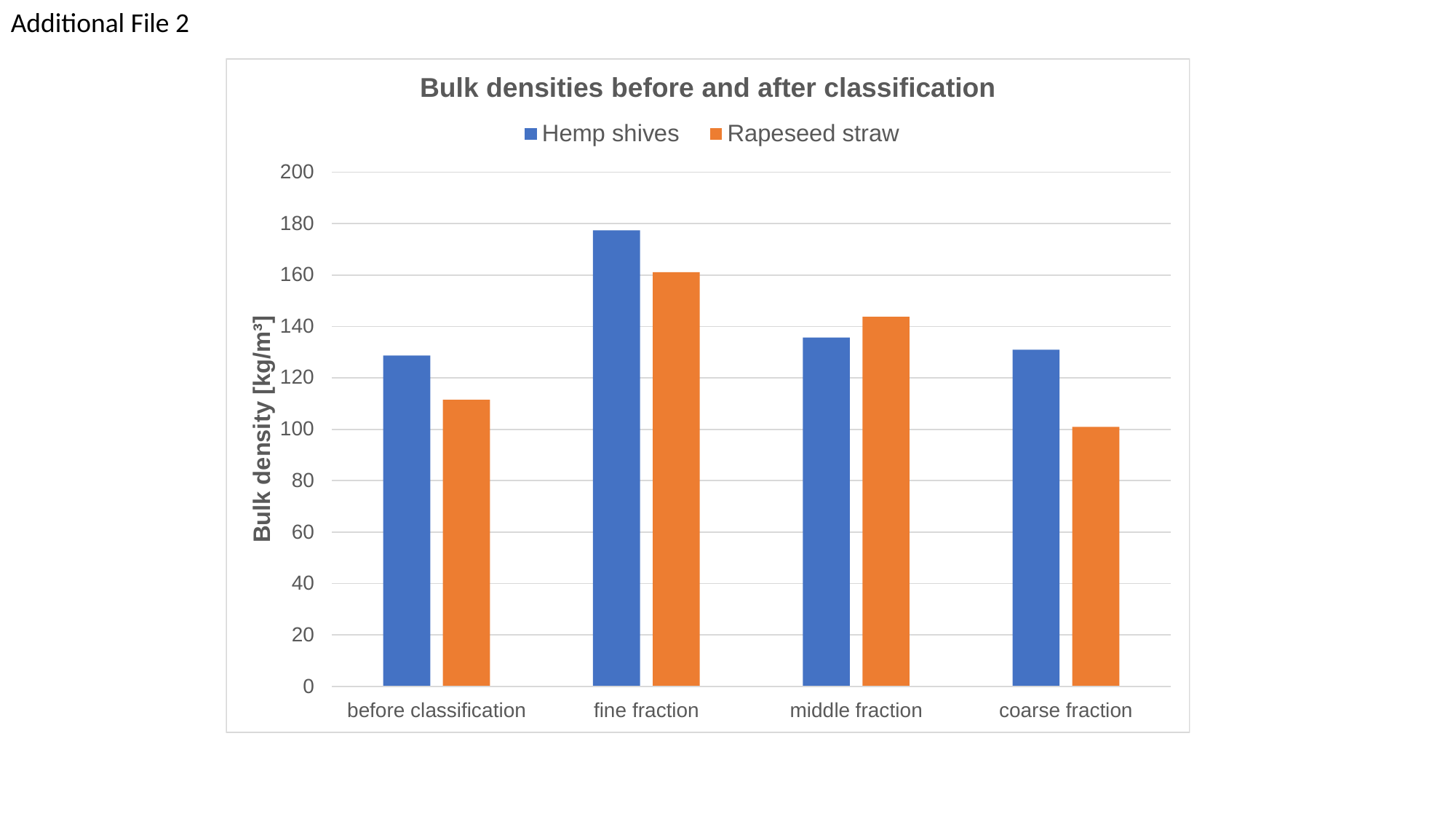

Additional File 2

Supplement: Supplementary file 2 — Additional file 2: Bulk densities of substrate fractions. [file 40694_2022_133_MOESM2_ESM.pptx]

## Slide 1
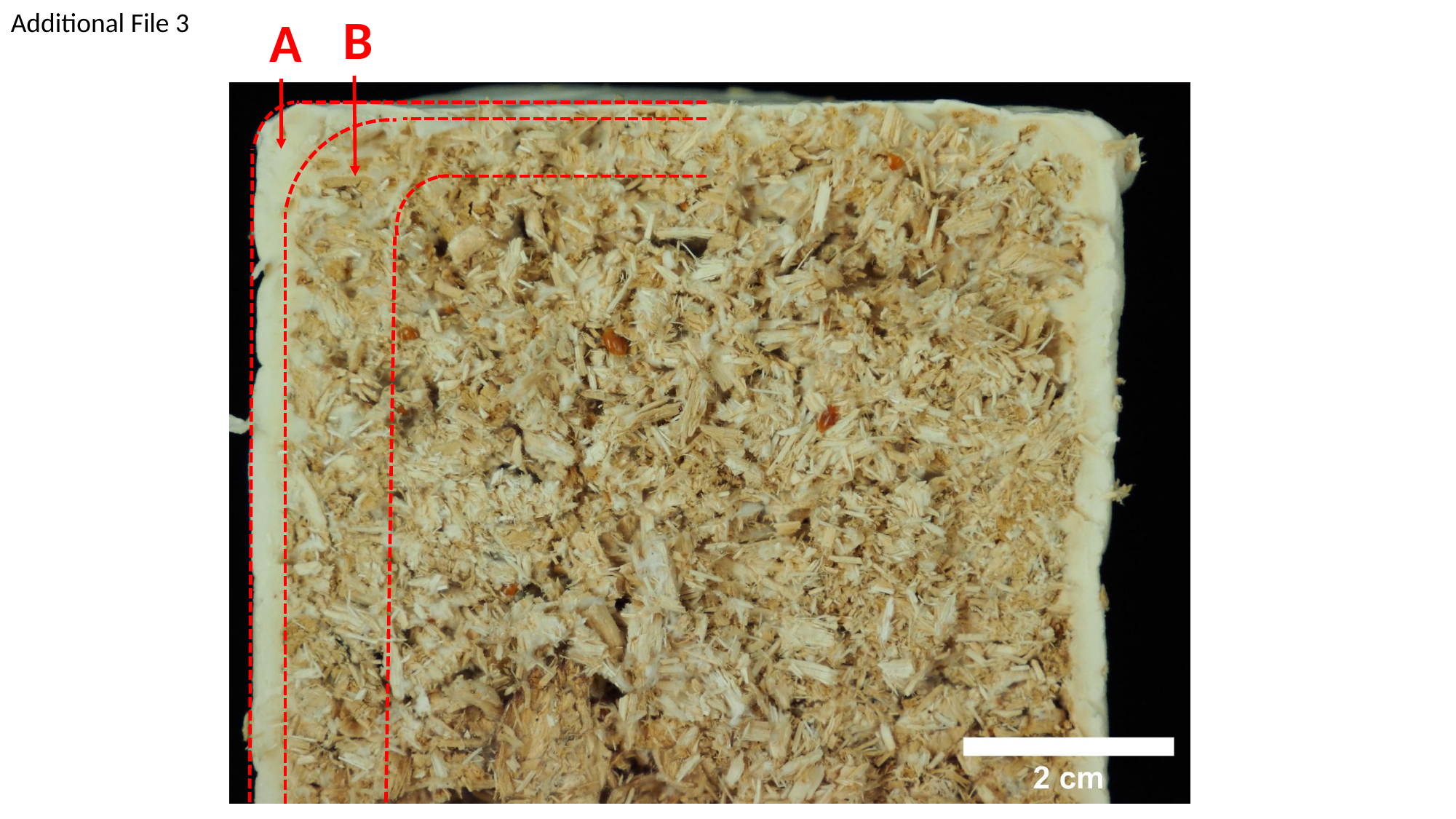

Additional File 3
B
A

Supplement: Supplementary file 3 — Additional file 3: Cut section of a composite. [file 40694_2022_133_MOESM3_ESM.pptx]

## Slide 1
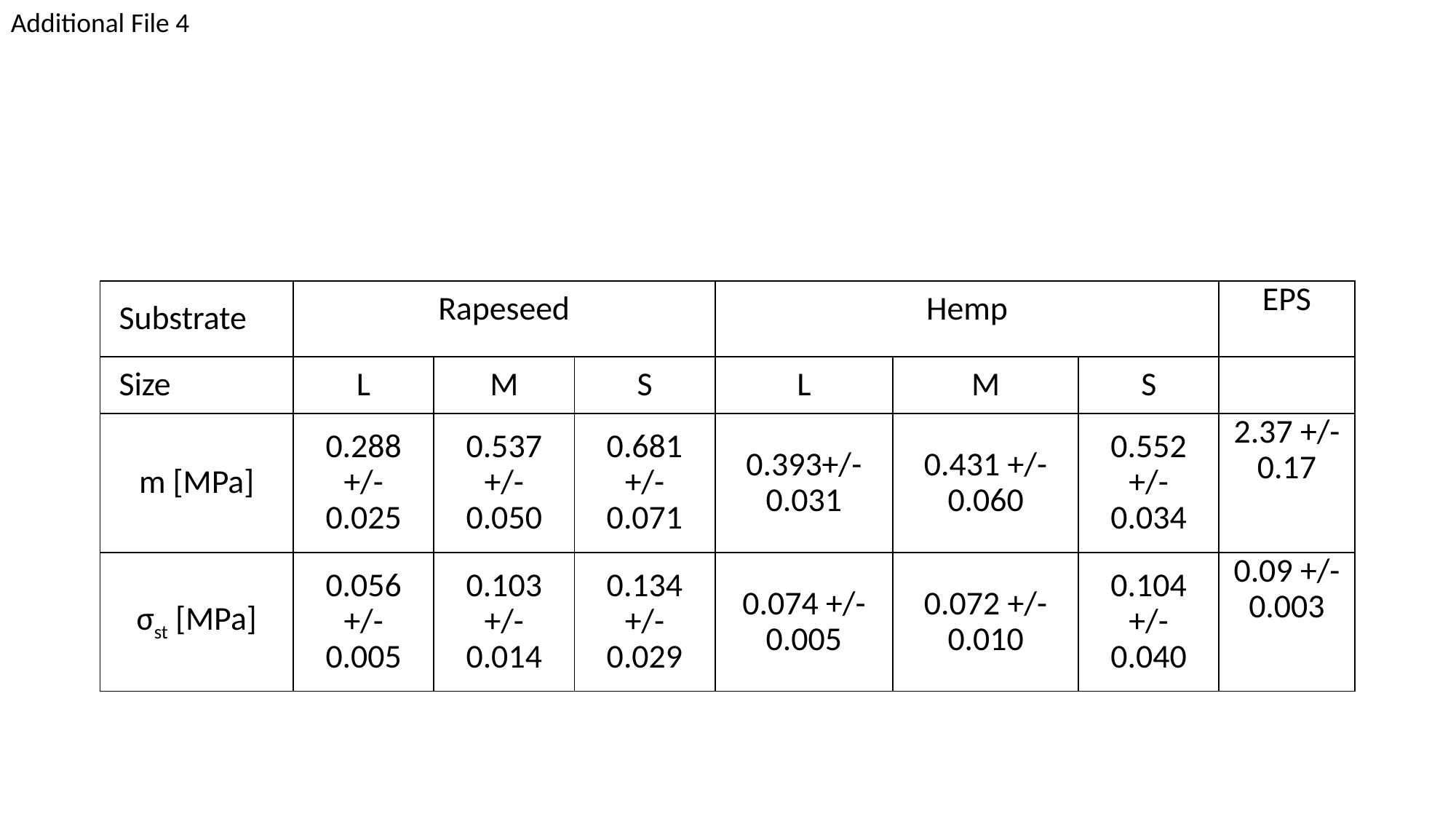

Additional File 4
| Substrate | Rapeseed | | | Hemp | | | EPS |
| --- | --- | --- | --- | --- | --- | --- | --- |
| Size | L | M | S | L | M | S | |
| m [MPa] | 0.288 +/- 0.025 | 0.537 +/- 0.050 | 0.681 +/- 0.071 | 0.393+/- 0.031 | 0.431 +/- 0.060 | 0.552 +/- 0.034 | 2.37 +/- 0.17 |
| σst [MPa] | 0.056 +/- 0.005 | 0.103 +/- 0.014 | 0.134 +/- 0.029 | 0.074 +/- 0.005 | 0.072 +/- 0.010 | 0.104 +/- 0.040 | 0.09 +/- 0.003 |

Supplement: Supplementary file 4 — Additional file 4: Elastic modulus and compression strengths of composites. [file 40694_2022_133_MOESM4_ESM.pptx]

## Slide 1
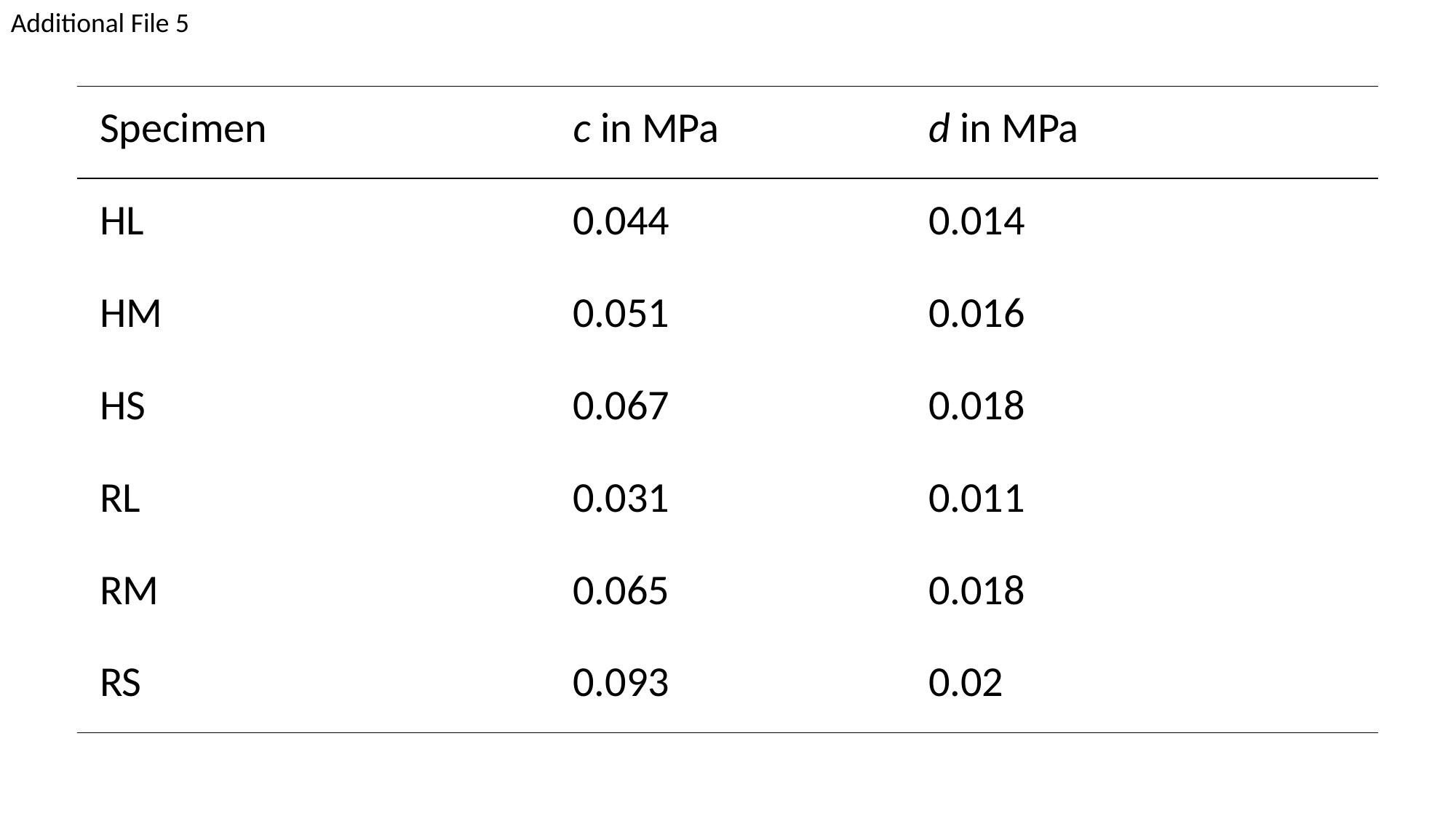

Additional File 5
| Specimen | c in MPa | d in MPa |
| --- | --- | --- |
| HL | 0.044 | 0.014 |
| HM | 0.051 | 0.016 |
| HS | 0.067 | 0.018 |
| RL | 0.031 | 0.011 |
| RM | 0.065 | 0.018 |
| RS | 0.093 | 0.02 |

Supplement: Supplementary file 5 — Additional file 5: Neo-Hookean material parameters. [file 40694_2022_133_MOESM5_ESM.pptx]

## Slide 1
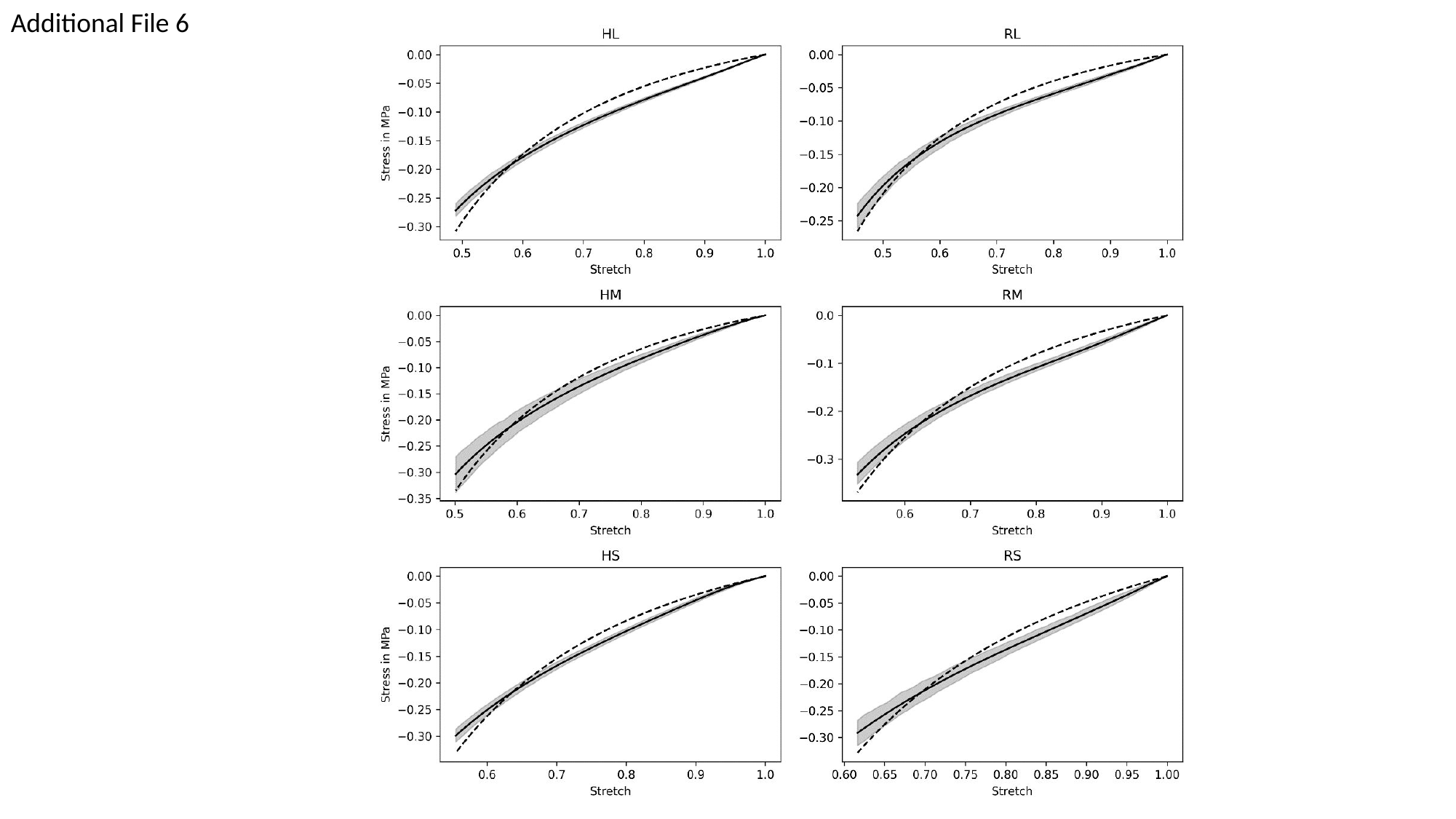

Additional File 6

Supplement: Supplementary file 6 — Additional file 6: Comparison of the Neo-Hookean model data with experimental data. [file 40694_2022_133_MOESM6_ESM.pptx]
